# A Taxpayer-Funded Clinical Trials Registry and Results Database

**DOI:** 10.1371/journal.pmed.0010060

**Published:** 2004-11-30

**Authors:** Erick H Turner

## Abstract

It already exists within the US Food and Drug Administration, argues a former clinical reviewer of psychotropic drugs at the FDA

Over the past several years, there has been growing concern about selective publication of clinical trial results [1,2]. The debate has intensified since New York State Attorney General Elliot Spitzer filed suit against GlaxoSmithKline on June 2, 2004, alleging that the company was hiding data regarding the efficacy and safety of selective serotonin reuptake inhibitors in pediatric patients with depression [3].

The two most frequently suggested remedies for the selective reporting of clinical trials results have been to register all clinical trials and to make their results publicly available. Registries have been called for at least as far back as 1974; hundreds have in fact already been established [4]. Shortcomings of registries include the fact that they are often not coordinated and that participation is often voluntary and—in cases where they are mandated by legislation—difficult to enforce. For example, ClinicalTrials.gov, a registry authorized by the Food and Drug Modernization Act of 1997, appears not to be comprehensive. One study found that, of 127 cancer protocols sponsored by pharmaceutical companies that met criteria for inclusion, only 48% were in fact submitted to the registry [5]. Thus, one can check a number of registries and still have little assurance that all the relevant trials of interest have been included.[Fig pmed-0010060-g001]

**Figure pmed-0010060-g001:**
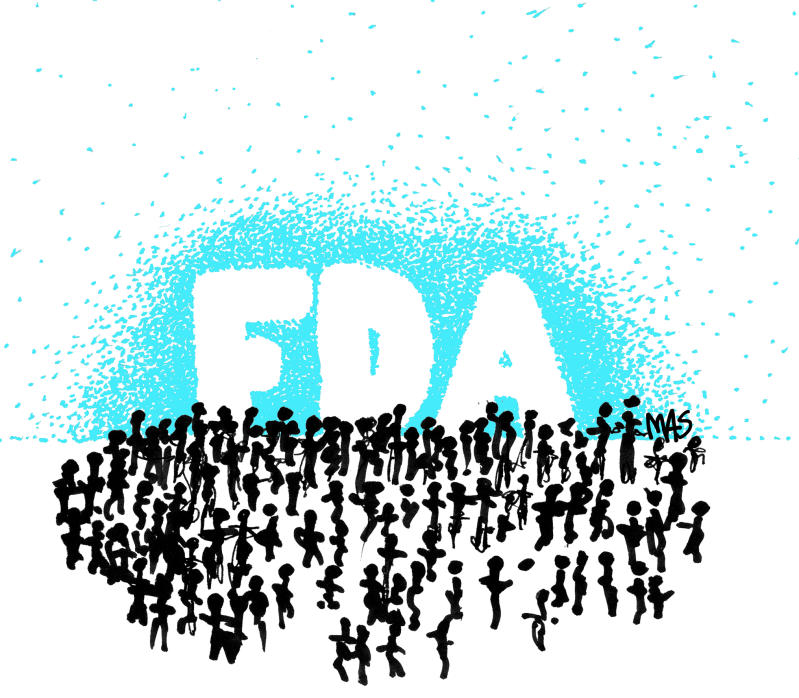
The public would benefi t from more freedom of information at the FDA (Illustration: Margaret Shear)

Increasing the pressure on pharmaceutical companies to include more trials in registries, the International Committee of Medical Journal Editors has announced that, as a condition of considering a trial for publication, member journals will require its registration in a public trials registry [6]. Further, at the American Medical Association (AMA) Annual Meeting of the House of Delegates in June 2004, the AMA called on the Department of Health and Human Services to establish a comprehensive national registry. In September 2004, an AMA trustee testified in a United States Congressional hearing, outlining elements necessary to make such a registry effective [7]. Momentum for a comprehensive clinical trials registry is also building internationally [8].

In this essay, I argue that a highly valuable but underused registry and results database for US trials already exists within the Department of Health and Human Services, specifically within the Food and Drug Administration (FDA).

## New Drug Applications

Before a pharmaceutical company can conduct a US trial that it intends to use in support of a new drug application (NDA), it must first register that trial with the FDA. Because the NDA forms the basis for marketing approval, it seems likely that the percentage of industry-sponsored trials that are registered with the FDA is very high. This registration takes the form of an investigational new drug (IND) application [9]. The IND contains a trial protocol; protocols for additional studies within the same clinical trials program are submitted as amendments to the IND. Later, when the sponsor has completed its clinical trials program and wishes to apply for marketing approval, it submits its NDA.

The FDA then begins the NDA review process, during which a physician, a statistician, and a pharmacologist, among others, generate lengthy review documents [10]. These reviews not only address the sponsor's analyses of the data on pivotal studies, but they often also include reanalyses by the reviewers using raw data obtained from the sponsor. These analyses are conducted in adherence to the statistical methods set forth a priori in the original trial protocols. (By contrast, with most journal publications, it is usually not possible for the reader to verify [Boxed-text box1]whether what is presented as the main finding is consistent with the original hypothesis or whether it was a post hoc finding.) After the primary reviewers have written their reviews, shorter reviews are written by their superiors, with the process culminating in a decision about whether to approve the drug for the proposed indication.

**Paroxetine for Anxiety Disorders: Checking the Published Literature against the FDA Reviews**

A Cochrane systematic review of antidepressants for generalized anxiety disorder [15] lists only one double-blind placebo-controlled study of paroxetine [16], a positive study. A PubMed search reveals no additional double-blind placebo-controlled studies. In accessing the review from Drugs@FDA (approval date April 2001), we learn that there were in fact three pivotal double-blind placebo-controlled studies. One of these studies corresponds to the published positive study noted above. Of the remaining two studies, both apparently unpublished, one was positive while the other was marginally positive.

Turning to the controlled-release formulation of paroxetine (Paxil CR) for panic disorder, a review article states in its abstract that the drug “demonstrated efficacy in three well designed studies in patients with panic disorder with or without agoraphobia” [17]. In reading the corresponding FDA statistical review, we verify that there were indeed three studies. However, the FDA statistical reviewer found that only one of these studies was strongly positive. A second study, judged “supportive” of efficacy, had a marginally significant (*p* = 0.039) result on a secondary observed-cases analysis, but a nonsignificant (*p* = 0.38) result on the primary efficacy analysis defined a priori. The third study was clearly negative, with p-values of 0.33 and 0.57 on the primary and secondary analyses, respectively.

## A Semi-Public Database

This process occurs entirely outside of the public domain. However, in the interest of making the FDA more “transparent,” and in accordance with the Electronic Freedom of Information Act [11], the FDA has, for the past several years, posted selected NDA reviews for approved drug–indication combinations on the FDA Web site Drugs@FDA (http://www.accessdata.fda.gov/scripts/cder/drugsatfda/index.cfm). These NDA review documents are much more detailed than the resulting package insert and often more detailed than corresponding journal publications.

For example, while the clinical trials section of the package insert is typically a few paragraphs long, the efficacy portion of the clinical review usually runs tens of pages. Because the FDA is made aware of all studies that the sponsor plans to use in support of the NDA before they are conducted, and thus before there can be any selection based on outcome, these reviews cover not only studies that are positive (and more likely to be published in journals), but also studies whose outcome was negative or indeterminate. The sidebar gives an example of how NDA review documents at the FDA give valuable information about paroxetine for anxiety disorders.

## FDA Reviews for All Approved Drugs Should Be Made Public

In the examples discussed in the sidebar, our having access to the FDA review documents allows us to become aware of, and see beyond, apparent publication bias. It is in the best interest of the public health for the FDA to make as many reviews available as possible. According to the FDA Web site, “As FDA continues to be one of the world's leading agencies in its emphasis on openness and transparency, it is aware that making even more information available to the public will further the Agency's mission to protect and promote public health and improve its credibility. For example, FDA has aggressively implemented the Electronic Freedom of Information Act…” [11].

Unfortunately, the availability of review documents on Drugs@FDA is sporadic. To take additional examples from psychiatry, NDA reviews have been posted on Drugs@FDA for some approved drug–indication combinations, such as fluoxetine for pediatric depression, and aripiprazole and quetiapine for schizophrenia. However, NDA reviews for many other drug–indication combinations have not been posted: the Prozac Weekly formulation of fluoxetine, clozapine for suicidal behavior in patients with schizophrenia or schizoaffective disorder, and quetiapine for mania, among others. A review on paroxetine for pediatric depression, the subject of Elliot Spitzer's suit against GlaxoSmithKline, is not posted. This is probably because this drug–indication combination was not approved; in fact, it is possible that GlaxoSmithKline did not file an NDA to be reviewed. However, I do not understand why, in cases where NDAs were both submitted and approved, such as the ones listed above, some reviews are posted while others are not.

I therefore suggest that we increase access to the clinical trials registry and results database that already exist within the FDA. The agency could expand its implementation of the Electronic Freedom of Information Act and make all NDA reviews, at least for approved NDAs, available in the public domain. The act is written into the FDA portion of the Code of Federal Regulations as follows: “The Food and Drug Administration will make the fullest possible disclosure of records to the public, consistent with the rights of individuals to privacy, the property rights of persons in trade secrets and confidential commercial or financial information” [12].

## Obstacles and Limitations

There would surely be obstacles. The pharmaceutical industry would vigorously invoke Exemption 4 of the Freedom of Information Act, the exemption for trade secrets and confidential business information [13]. However, the FDA Freedom of Information Office already deals with confidential and proprietary information by redacting or editing it out of the review documents before making them available. Within the FDA's Freedom of Information Office, staffing would need to be greatly increased. Some oversight might be necessary to ensure that the taxpaying public has been granted the fullest possible access and that unwarranted redaction does not occur. Unless the Freedom of Information Act is modified, access would still likely be limited to approved NDAs. Data would remain unavailable for trials that did not lead to an approved NDA.

It should be clarified that this resource does not compete with proposals by the AMA and other groups for clinical trial registries—rather, it complements them. The AMA has proposed the creation of a registry that is comprehensive in scope. The FDA's registry and results database are restricted to those trials aimed at supporting US marketing approval or a change in labeling in the US. While data from many studies conducted abroad are submitted to the FDA for this purpose, this is not the case for drugs for which the sponsor has elected not to seek approval for marketing in the US. Nor does the FDA review data from most trials funded by other US government agencies, such as the National Institutes of Health, or by foundations. And drug companies fund investigator-initiated trials that are often not registered with the FDA.

To make the FDA review data more accessible and user-friendly, simple formatting changes would be needed. For those (few) reviews that are currently posted on Drugs@FDA, one can determine the indication being evaluated only after opening the document and paging through it. (Descriptive titles would be helpful, and these could be linked to ClinicalTrials.gov. Further, the trials reviewed could be identified with a unique international identifier, as promoted by the World Health Organization [14].) Despite the fact that the reviews are created in Microsoft Word and converted to PDF, the versions that appear on the Web site are no longer in a searchable text format. While the reviews tend to be well organized, the posted versions are difficult to navigate because there is no hyperlinked table of contents. In addition to having these formatting issues addressed, clinicians and patients might benefit from brief summaries, the writing of which might require the addition of new FDA staff.

## Conclusion

Despite the limitations of the FDA's database, making it public is a strategy that could be implemented both rapidly and easily by building upon existing infrastructure. While we await the creation of a clinical trials registry and results database that is truly comprehensive, we already have at our disposal one that could serve as a trove of in-depth and unbiased information on many, if not most, drugs currently marketed in the US.

## Abbreviations

AMA: American Medical Association
FDA: Food and Drug Administration
IND: investigational new drug
NDA: new drug application
